# Post myocardial infarction left ventricular intramural dissecting hematoma: a case report describing a very rare complication

**DOI:** 10.1186/s12872-022-02523-x

**Published:** 2022-03-04

**Authors:** Hitesh Gurjar, Muhammad Saad, Nisha Ali, Sudiksha Regmi, Prakash Upreti, Sumit Karna, Sandhya K. Balaram, Gayathri Kamalakkannan, Timothy J. Vittorio

**Affiliations:** 1 Department of Internal Medicine, BronxCare Hospital Center, Selwyn Avenue, Apt 17-H, Bronx, NY 165010457 USA; 2 Icahn School of Medicine at Mount Sinai, New York, NY 10457 USA; 3 Department of Internal Medicine and Division of Cardiology, BronxCare Hospital Center, 1650, Selwyn Avenue, Apt 17-H, Bronx, NY 10457 USA; 4 Department of Cardiothoracic Surgery, Mount Sinai Hospital, Icahn School of Medicine at Mount Sinai, New York, NY 10457 USA

**Keywords:** Left ventricular intramural dissecting hematoma (LV-IDH), Coronary artery disease (CAD), Mechanical complication, Post myocardial infarction complication, Contained left ventricular rupture

## Abstract

**Background:**

Dissecting intramural hematoma is a rare complication of acute myocardial infarction (AMI) and has been associated with increased mortality. There has been paucity of literature to establish protocols and guidelines for management in such cases.

**Case presentation:**

We hereby report the case of a 45-year-old male patient with left ventricular intramural dissecting hematoma (LV-IDH) who presented with chest pain and breathlessness and diagnosed as non-ST-elevation myocardial infarction (NSTEMI). Transthoracic echocardiography (TTE) was performed showing LV-IDH, confirmed with cardiac magnetic resonant imaging (cMRI). Selective coronary arteriography (CAG) was performed showing significant obstructive coronary artery disease (CAD). Further management with conservative approach involved discussion with patient, cardiothoracic surgeon and cardiology team including heart failure specialist and interventional cardiology.

**Conclusions:**

This case describes a rare complication of AMI and also focuses on utility of TTE and cMRI in the diagnosis of this rare complication. Both diagnosis and management are challenging and have to be individualized in similar cases. Multidisciplinary care coordination is important in management of patients with this diagnosis.

**Supplementary Information:**

The online version contains supplementary material available at 10.1186/s12872-022-02523-x.

## Background

Intramural dissecting hematoma (IDH) is a rare complication after AMI. It has been variously termed as intramural dissecting hematoma, intramural hematoma, intramyocardial dissecting hematoma in literature. It can also occur spontaneously after percutaneous coronary intervention (PCI) or chest trauma and can extend in to left ventricular (LV) free wall, interventricular septum, or the right ventricle. It can be considered to be a part of the spectrum between ventricular septal and free wall rupture where the dissection plane does not extend to epicardium, but rather extends into myocardium. However, similar to ventricular septal rupture, it carries high mortality and treatment options can vary between conservative management to surgical evacuation and LV repair. Management of IDH must be individualized based on size of the hematoma, LV function and clinical and hemodynamic status of the patient, after detailed discussion with the patient.

## Case presentation

A 45-year-old male who presented to the emergency department (ED) with one month history of intermittent chest pain worsening for one day before presentation. He also had associated breathlessness on exertion for the same duration. His past medical history was significant for diabetes mellitus, hypertension, dyslipidemia, active cigarette smoking and established ischemic heart disease (IHD) with CAD for which he required PCI four years before presentation. He had family history of premature CAD. He denied any other recreational drug use. He has history of medication non-adherence and has not used any medications in past two years including the recommended antiplatelet agents, statins, anti-hypertensives, and anti-diabetic medications.

In the ED, patient continued to have anginal chest pain. He was diaphoretic and had breathlessness at rest. The temperature was 98.6-degree Fahrenheit, blood pressure 162/122 mmHg, heart rate 100 beats per minute, respiratory rate 16 per minute and oxygen saturation 99% on room air. On physical examination he appeared anxious, diaphoretic and tachypneic. Cardiopulmonary examination showed raised jugular venous pressure, mild pedal edema and crepitations involving less than one-third of both lung fields. Rest of the physical examination was unremarkable. His 12-lead electrocardiogram (ECG) showed sinus tachycardia and right bundle branch block (RBBB) with borderline ST-segment elevation in precordial V4 and V5 leads with q waves in inferior leads II, III, aVF as shown in Additional file 1: Fig. 1. He received loading dose of aspirin, clopidogrel, and unfractionated heparin in ED.

The initial laboratory findings in ED were significant for hemoglobin of 14.7 g per deciliter (reference range 12.0–16.0), white blood cell count of 13,300 per cubic millimeter (reference range 4800–10,800). The serum creatinine was 1.9 mg per deciliter (reference range 0.5–1.5), and troponin level was 349 nanogram per liter (reference range, < 12) which increased to 417 nanogram per liter over four hours. The hemoglobin A1C value was 6.9% (reference range 4.7–6.4). The Pro-B-Type-natriuretic peptide (ProBNP) level was 25,951 picogram per milliliter (reference range 0–125).

He was admitted in cardiac care unit (CCU) with the diagnosis of NSTEMI and started on continuous intravenous infusion of unfractionated heparin in accordance with NSTEMI management guidelines. He was also started on intravenous diuretics. The TTE showed reduced LV ejection fraction and dyskinesis in left anterior descending (LAD) territory in apical region. The LV apex showed echogenic lucent area with endomyocardial border as shown in Additional file 1: Fig. 2. There was no communication with LV cavity, pericardial space or right ventricular (RV) when assessed with DEFINITY ® (perflutren lipid microsphere) contrast and color doppler as shown in Additional file 1: Fig. 3. His anti-platelet agents and heparin infusion were held, and urgent cardiothoracic consult was obtained. The CAG was performed which showed critical disease with 70% stenosis of distal left main (LM) coronary artery, chronic total occlusion (CTO) of right coronary artery (RCA) and LAD (after septal branch with faint distal flow from collaterals), and 80% stenosis of left circumflex coronary artery (LCX).

The cMRI was done for viability assessment and showed transmural infarct involving 49% of the LV mass involving inferior, apical, mid-ventricular septum (anterolateral and inferolateral), inferolateral and anterior walls. It confirmed large intramural hematoma involving apical segments with myocardial rupture involving epicardium but with intact endocardium with viable tissue in LCX territory and partially viable tissue in LAD territory as shown in Additional file 1: Fig. 4. Due to the extent of hematoma and hemodynamic stability, patient was planned for conservative management with plan for follow up imaging for LV-IDH, and medical management for IHD with CAD. He was monitored for 1 week in hospital with follow up TTE showing stable hematoma. He was then discharged on maximally tolerated medical management including high intensity statins, single antiplatelet agent (aspirin), beta blockers, sacubitril-valsartan, nitrates, and diuretics and wearable external defibrillator for close follow up in clinic. He was counselled for smoking cessation. He underwent repeat TTE after discharge which showed LV-IDH was still stable in size. Treatment options were further reviewed with patient by a multidisciplinary team, and he was referred for cardiac transplantation.

## Discussion and conclusion

The LV-IDH is a rare and dreaded mechanical complication that occurs after AMI. In majority of cases, hematoma involves LV, followed by left atrial wall (after ablation procedures) [1], or RV [2]. The most common primary etiology of IDH is AMI, however, other causes such as spontaneous occurrence [3], post aortic root surgery [4], takotsubo cardiomyopathy [5], trauma [6] have been described.

In a necropsy study by Vargas-Barŕon et al., septum was the most common region involved followed by the LV free wall. Anterior wall AMI accounts for majority of the cases, with only small percentage contributed by inferior wall AMI [7]. Most cases occur in female patients, after the first episode of AMI and are usually associated with single vessel involvement along with absence of collateral vessel flow.

The IDH comprises of myocardium, epicardium and pericardium in its outer wall and myocardium and endocardium in its inner wall facing ventricular cavity. The pathophysiologic mechanism of IDH is dissection of myocardial tissue contributed by poor support from infarcted tissue, remodeling of small vessels in infarcted tissue, and raised intra-cavitary pressure.

The ECG may show bundle branch blocks in more than half of cases and may show persistent ST-segment elevation. TTE remains the cornerstone of diagnosis as in our case. It can show varying patterns of IDH, in varying stages of resorption, and formation of thrombus. Our case also showed intramural areas of resorption reflected by significant echo-lucent regions within IDH. Echocardiographic features include a thin layer of freely mobile endocardium or myocardium on one side and pericardium along with thicker layer of myocardium on the other side with doppler demonstration of color flow in the cavity. Occasionally entry point can be seen as in our case, where entry point was the junction of normal and infarcted IVS. Other imaging modalities such as cardiac MRI are especially useful as LV cavitary thrombus and LV aneurysm can closely mimic IDH (see Additional file 1: table 01) [8].

Overall mortality has been reported to be 47% with higher mortality reaching up to 78% in cases involving IVS [7]. Treatment options include conservative management to allow resorption of hematoma versus surgical evacuation and LV repair. Treatment decision can be made based on hemodynamic stability, extent of hematoma and LV dysfunction. Due to the lack of established treatment protocols, it may be reasonable to opt for conservative management with serial imaging follow up in clinically stable patients. The risk of surgical mortality is high especially with cardiopulmonary bypass, clot retraction and ventricular wall repair. Markers of poor prognosis include cardiogenic shock, ventricular septal rupture, LV dysfunction and pericardial effusion. Ventricular arrhythmias are common in these patients with risk of sudden cardiac death and should be carefully monitored. Spontaneous resolution was observed in majority of the cases reported in literature especially those involving the LV apex. Multidisciplinary team approach should be adopted in such cases. In current case hemodynamic stability, non-viability on cMRI and high surgical mortality risk, played major role in opting for conservative approach initially followed by cardiac transplant referral.

In summary, LV-IDH is a rare mechanical complication of myocardial infarction. Contrast enhance echocardiogram remains the cornerstone of diagnosis with differentials including LV thrombus or aneurysm. Cardiac MRI is a useful adjunct to assess for extent of IDH and rule out other differentials. Given lack of established treatment guidelines, management include individualized approach as both conservative management expecting resorption of hematoma, as well as surgical evacuation of hematoma have been described. Cardiac transplantation can also be considered in patients at high risk for surgical complications.

## Supplementary Information

**Additional file 1. Table-01:** Differential diagnosis of IDH and echocardiographic and MRI features. **Figure 1:** Electrocardiogram showing sinus tachycardia, qRBBB pattern and borderline ST segment elevation in V4 and V5 leads. **Figure 2:** a) figure shows echocardiogram in parasternal long axis view demonstrating echo lucent space suggesting intramural hematoma involving apical region with entry point along interventricular septum. b) echocardiogram in parasternal short axis view (angulated to include maximum width of IDH) showing intramural hematoma, in apical segments extending from mid cavity level. **Figure 3:** a) Echocardiogram in parasternal short axis view shows no flow across endocardium to intraventricular region. b) Echocardiogram with perflutren lipid microsphere (DEFINITY ®) injectable suspension contrast agent showing no communication between LV cavity and intramural space. **Figure 4:** Cardiac MRI showed intramural dissecting hematoma with communication with the LV apex.

## Data Availability

Data and related clinical information can be obtained from corresponding author.

## Abbreviations

AMI: Acute myocardial infarction
NSTEMI: Non-ST-segment elevation myocardial infarction
cMRI: Cardiac magnetic resonant imaging
IDH: Intramural dissecting hematoma
LV: Left ventricular
LCX: Left circumflex coronary artery
RCA: Right coronary artery
LAD: Left anterior descending
CTO: Chronic total occlusion
LM: Left main
TTE: Transthoracic echocardiogram
ProBNP: Pro-B-Type natriuretic peptide
CAD: Coronary artery disease
IHD: Ischemic heart disease
ED: Emergency department
LV-IDH: Left ventricular intramural dissecting hematoma
