# Supplementary material for: Post myocardial infarction left ventricular intramural dissecting hematoma: a case report describing a very rare complication
Source: BMC Cardiovasc Disord. 2022 Mar 4;22:83. doi: 10.1186/s12872-022-02523-x (PMC8895780; doi:10.1186/s12872-022-02523-x)
Supplement: Supplementary file 1 — Additional file 1. Table-01: Differential diagnosis of IDH and echocardiographic and MRI features. Figure 1: Electrocardiogram showing sinus tachycardia, qRBBB pattern and borderline ST segment elevation in V4 and V5 leads. Figure 2: a) figure shows echocardiogram in parasternal long axis view demonstrating echo lucent space suggesting intramural hematoma involving apical region with entry point along interventricular septum. b) echocardiogram in parasternal short axis view (angulated to include maximum width of IDH) showing intramural hematoma, in apical segments extending from mid cavity level. Figure 3: a) Echocardiogram in parasternal short axis view shows no flow across endocardium to intraventricular region. b) Echocardiogram with perflutren lipid microsphere (DEFINITY ®) injectable suspension contrast agent showing no communication between LV cavity and intramural space. Figure 4: Cardiac MRI showed intramural dissecting hematoma with communication with the LV apex. [file 12872_2022_2523_MOESM1_ESM.docx]

**Table-01: Differential diagnosis of IDH and echocardiographic and MRI features**

| **LV Pseudoaneurysm** | Complete rupture of myocardium lined by pericardium | Dyskinetic segment with focal bulging of the pericardium. |
| --- | --- | --- |
| **LV thrombus** | Intracavitary thrombus with identification of endocardium and systolic expansion | Dark mass with strong enhancement of the LV cavity |
| **LV noncompaction** | Prominent trabeculations with color flow within the trabeculations. | An end-diastolic ratio between non-compacted and compacted layers of greater than 2. |
| **LV IDH** | Thin layer of freely mobile endocardium on one side and thicker layer of myocardium on the other side with doppler color flow in the cavity. | Intramyocardial dissection cavity containing the hematoma |


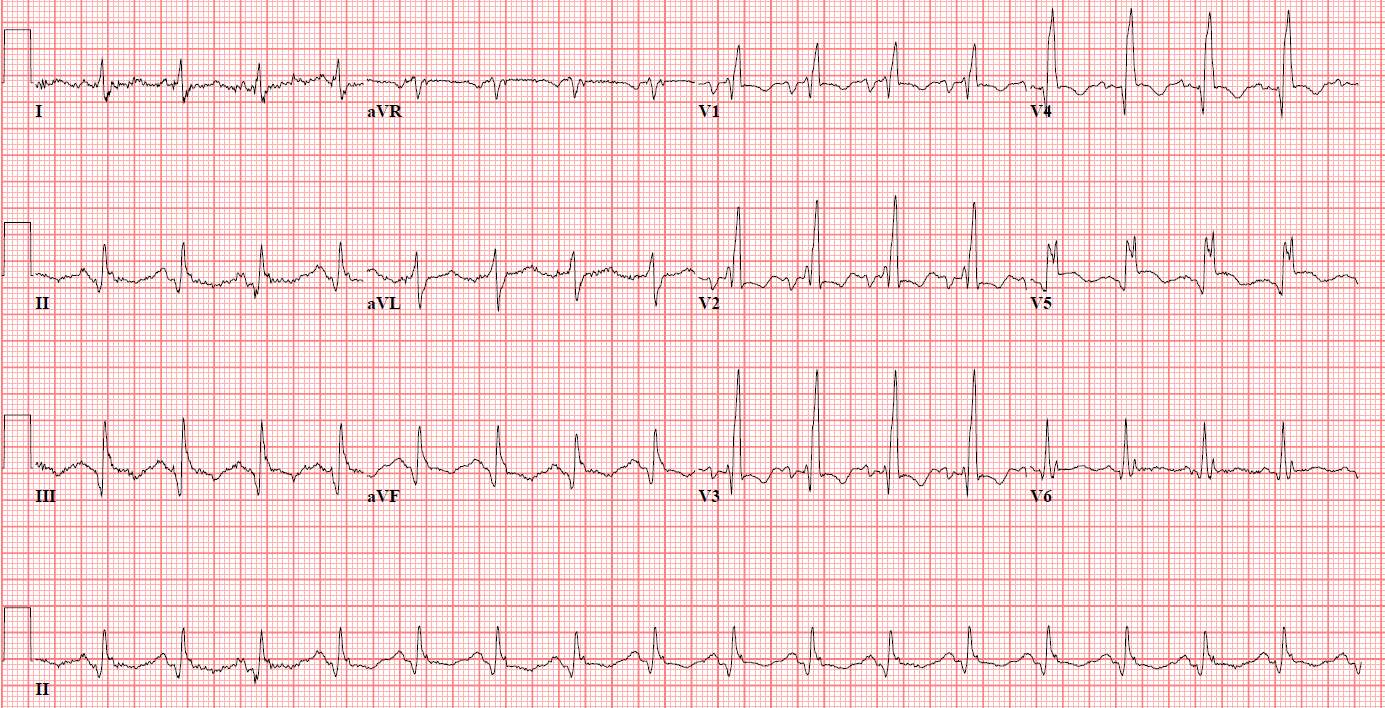
**Figure 1**: Electrocardiogram showing sinus tachycardia, qRBBB pattern and borderline ST segment elevation in V4 and V5 leads.


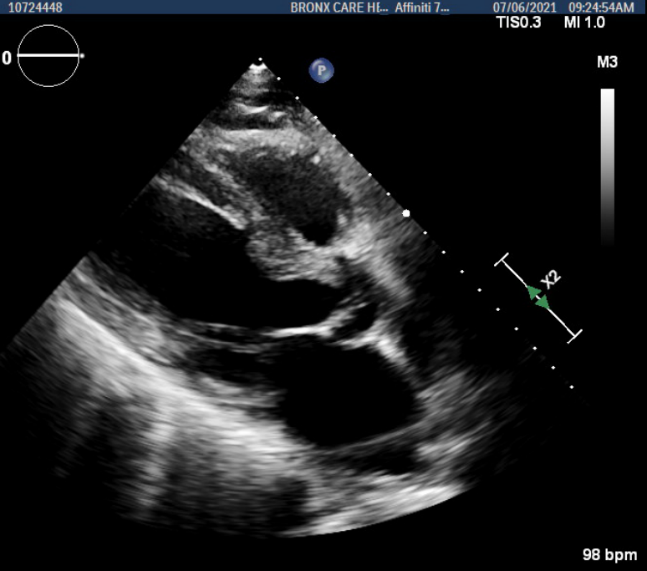

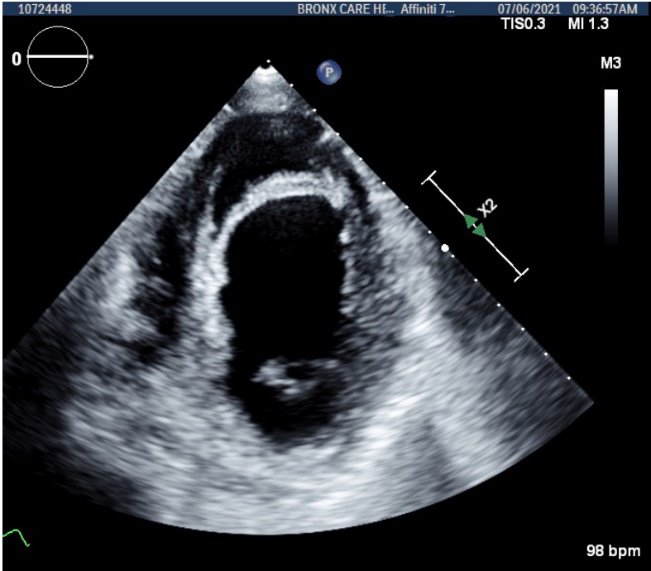


**Figure 2**: a) figure shows echocardiogram in parasternal long axis view demonstrating echo lucent space suggesting intramural hematoma involving apical region with entry point along interventricular septum. b) echocardiogram in parasternal short axis view (angulated to include maximum width of IDH) showing intramural hematoma, in apical segments extending from mid cavity level.


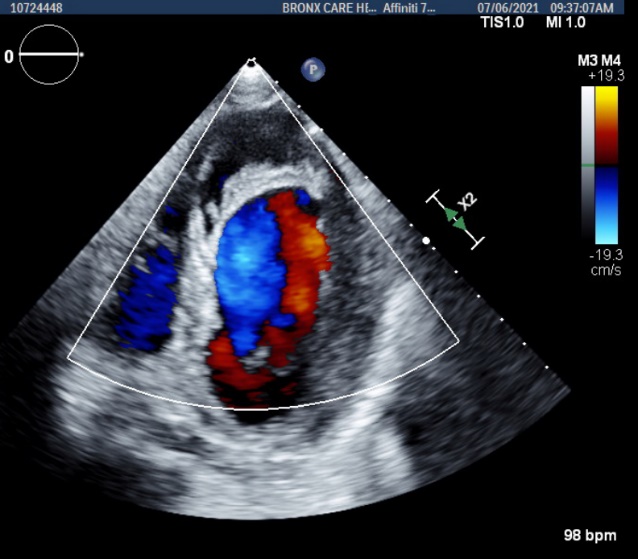

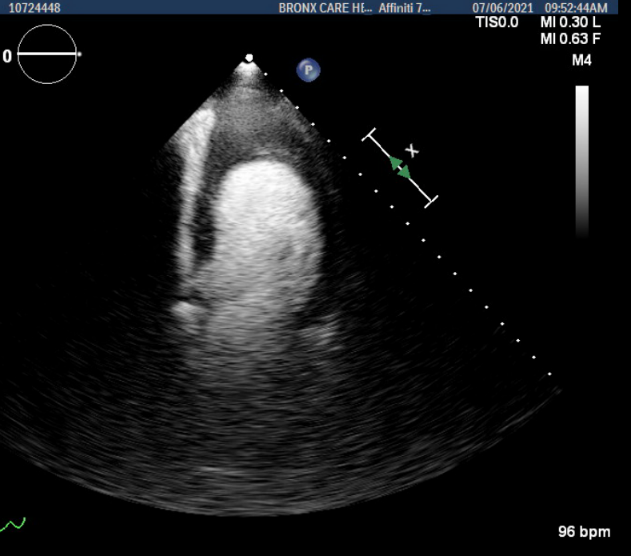


**Figure 3**: a) Echocardiogram in parasternal short axis view shows no flow across endocardium to intraventricular region. b) Echocardiogram with perflutren lipid microsphere (DEFINITY ®) injectable suspension contrast agent showing no communication between LV cavity and intramural space.


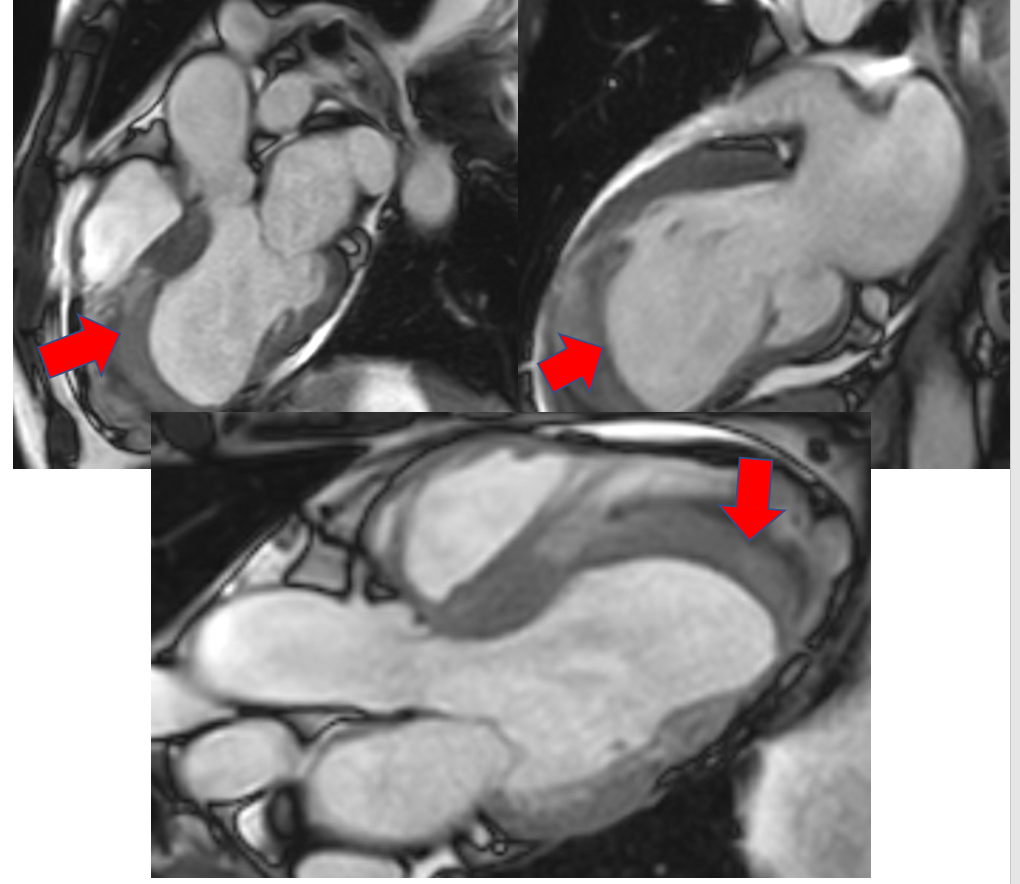
**Figure** **4**: Cardiac MRI showed intramural dissecting hematoma with communication with the LV apex.
